# Pituitary Apoplexy Presenting as Ophthalmoplegia and Altered Level of Consciousness without Headache

**DOI:** 10.1155/2018/7124364

**Published:** 2018-05-09

**Authors:** Nooshin Salehi, Anthony Firek, Iqbal Munir

**Affiliations:** ^1^Department of Medicine, Riverside University Health System Medical Center, Moreno Valley, CA, USA; ^2^Division of Endocrinology, Department of Medicine, Riverside University Health System Medical Center, Moreno Valley, CA, USA

## Abstract

*Background. *Pituitary apoplexy (PA) is a clinical syndrome caused by acute ischemic infarction or hemorrhage of the pituitary gland. The typical clinical presentation of PA includes acute onset of severe headache, visual disturbance, cranial nerve palsy, and altered level of consciousness.* Case Report*. A 78-year-old man presented to the emergency department with one-day history of ptosis and diplopia and an acute-onset episode of altered level of consciousness which was resolving. He denied having headache, nausea, or vomiting. Physical examination revealed third-cranial nerve palsy and fourth-cranial nerve palsy both on the right side. Noncontrast computed tomography (CT) scan of the head was unremarkable. Brain magnetic resonance imaging (MRI) showed a pituitary mass with hemorrhage (apoplexy) and extension to the right cavernous sinus. The patient developed another episode of altered level of consciousness in the hospital. Transsphenoidal resection of the tumor was done which resulted in complete recovery of the ophthalmoplegia and mental status.* Conclusion*. Pituitary apoplexy can present with ophthalmoplegia and altered level of consciousness without having headache, nausea, or vomiting. A CT scan of the head could be negative for hemorrhage. A high index of suspicion is needed for early diagnosis and timely management of pituitary apoplexy.

## 1. Background

Pituitary apoplexy (PA) is a potentially life-threatening disorder caused by acute ischemic infarction or hemorrhage of the pituitary gland [1]. Pituitary adenomas are prone to bleeding and necrosis, possibly because they outgrow their blood supply or because tumor expansion causes ischemia and resultant infarction by compressing the vessels against the sellar diaphragm [2]. The inherent fragility of tumor blood vessels may also explain the tendency to hemorrhage [2]. However, PA may also occur in nonadenomatous or even the normal pituitary gland, especially during pregnancy [1]. In one report, the prevalence of pituitary apoplexy among patients with nonfunctioning pituitary macroadenomas was 8 percent [3].

The clinical presentation of PA is highly variable and is largely determined by the extent of hemorrhage, necrosis, and edema. Headache of sudden and severe onset is the main symptom of PA and is present in more than 80% of the patients [2]. Patients can also present with visual disturbances, diplopia, hypopituitarism, and impaired consciousness which, together with the radiological evidence of a pituitary lesion, establish the diagnosis [4]. In cases with severe, progressive visual, or neurological manifestations, surgical decompression is indicated [4], while the patients with mild stable clinical picture can be managed conservatively [4].

PA may present as isolated acute cranial nerve (CN) palsies [5–11], which usually indicates a milder disease [4] and possibly a more chronic onset. In the setting of PA, the third cranial nerve (CN III) is the most frequently affected cranial nerve [4–7, 10], but sixth-cranial nerve (CN VI) palsy [12], multiple CN palsies [8, 13], and even bilateral lesions [9, 14] have also been reported. CT scan of the head typically shows hemorrhage in the pituitary gland. Pituitary apoplexy presenting with ophthalmoplegia and recurrent brief episodes of altered level of consciousness, without demonstrating headache, nausea, or vomiting has not been previously described in the literature. The following is an illustrative case of an unusual presentation of PA in an elderly man and a focused review of the literature.

## 2. Case Report

A 78-year-old African American man with past medical history of hypertension and alveolar emphysema presented to the emergency department with altered level of consciousness (LOC). He was found unresponsive and drooling on the couch by a family member. The patient's family had noted drooping of the right eyelid that had started the day before the incident. No headache, head injury, chest pain, palpitation, or decreased vision was reported but it was noted that he possibly could not see well in his peripheral visual field. The emergency medical services (EMS) reported an initial Glasgow Coma Score (GCS) of 7/15 (Eye 1, Verbal 1, and Motor 5) that began to improve on the way to the hospital. According to the EMS report, the patient's vital signs were normal at the scene including a blood pressure of 118/82 mmHg, heart rate of 92 per minute, and respiratory rate of 12 per minute. His blood glucose level was at 155 mg/dL.

He was a smoker, using a quarter of pack of cigarettes a day. His medications included amlodipine, ipratropium nebulizer, and albuterol nebulizer. He did not use alcohol or illicit drugs.

Upon arrival to the hospital, the patient was lethargic and had poor attention but was oriented to the time, place, and person. He denied headache, nausea, or vomiting. Physical examination revealed a GCS of 14/15 and normal vital signs, including blood pressure of 115/76 mmHg, body temperature of 37°C, respiratory rate of 20 per minute, and a regular pulse rate of 83 beats per minute. Pupils were equal in size and both had normal direct and consensual responses to light. Visual acuity appeared to be reduced but full evaluation was not possible. Complete ptosis of the right eye was noted. The right eye was deviated to the right with severe limitation in supraduction, infraduction, and adduction consistent with pupil-sparing third-cranial nerve (CN III) palsy. Fourth-cranial nerve (CN IV) palsy was also present in the right side. Extraocular eye movements were intact in the left eye. Sensation was intact over the face bilaterally. There were no other abnormal findings in the physical exam. Upon the admission, the plasma sodium level was slightly depressed at 130 mEq/L but the other initial lab findings were unremarkable (Table 1).

**Figure 1:**
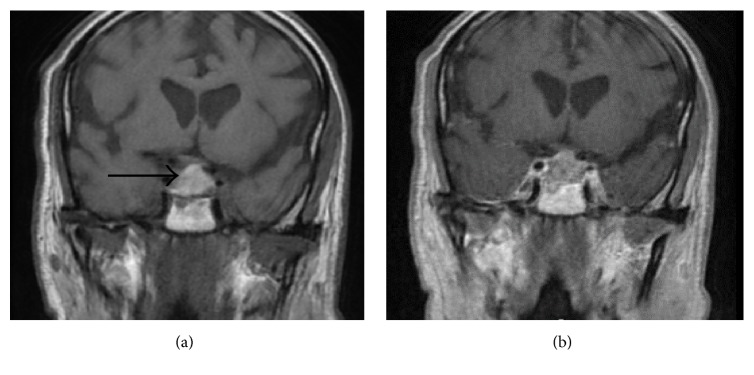
Brain MRI W/WO contrast 2 days before surgery. (a) Coronal T1-weighted image demonstrates a large sellar mass with suprasellar extension and slight mass effect on the optic chiasm. This mass exhibits marked hyperintense signal on T1-weighted image (arrow), which indicates blood products and recent hemorrhage. (b) Coronal T1-weighted postcontrast image shows normal enhancement of the cavernous sinus. No enhancing mass or nodule is evident in association with intrinsically T1 bright lesion. The mass abuts approximately 90 degrees of the right internal carotid artery contour. This abutment is less on the left side. The carotid arteries are patent and show normal caliber without any narrowing. No normal pituitary tissue is identified.

The patient was admitted to the internal medicine service for a stroke workup. Noncontrast computed tomography scan (CT scan) of the head showed only age related atrophic changes with no evidence of intracranial hemorrhage or mass effect. Brain magnetic resonance imaging (MRI) showed a sellar mass, and the pituitary MRI (Figure 1) revealed a large pituitary mass with extension to the right cavernous sinus. Intrinsic T1 hyperintensity suggested possible hemorrhage (pituitary apoplexy). Mild mass effect on the optic chiasm was noted. There were no evidence of extension of the mass beyond the lateral confines of the cavernous sinuses and no hydrocephalus or subarachnoid hemorrhage. The major intracranial flow voids were patent.

Lumbar puncture was performed and cerebrospinal fluid (CSF) analysis did not show any blood or xanthochromia. On his second hospital day, the patient developed an acute change in the mental status. He was noted to be lethargic and confused, not responding to his name, and having garbled speech. During that time, he had blood sugar of 98, blood pressure of 98/56 mmHg, and heart rate of 113 per minute. His acute confusional state resolved within an hour, but a clouding of consciousness continued to be present as was observed since admission. Electrocardiogram (EKG) and echocardiography were normal.

Based on the ophthalmoplegia, ptosis, altered mental status, visual symptom, and confirmatory findings in the MRI, the diagnosis of pituitary apoplexy was established. The hormonal profile (Table 1) included a low serum cortisol at baseline (1.1 mcg/dL) and low concurrent adrenocorticotropin hormone (ACTH). Cosyntropin stimulation increased the cortisol to 11.6 mcg/dL after 60 minutes. Free thyroxine (T4) and thyroid stimulating hormone (TSH) were normal but prolactin was below the normal range. Insulin-like growth factor 1 (IGF-1), follicular stimulating hormone (FSH), and luteinizing hormone (LH) were normal.

**Figure 2:**
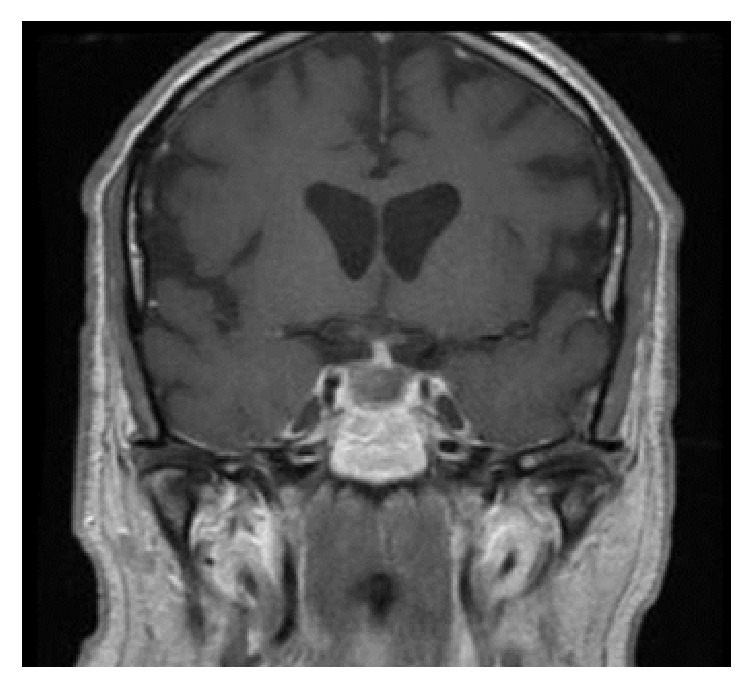
MRI (T1 weighted with contrast) one day after the surgery shows interval resection of the previously seen sellar mass/hemorrhage and resolution of mass effect on the optic chiasm.

After the blood was collected for the endocrine workup, intravenous hydrocortisone was administered with the dose of 100 mg every 8 hours for 36 hours and then transitioned to oral hydrocortisone 20 mg in the morning and 10 mg at noon. On the fourth hospital day, transnasal transsphenoidal resection of the sellar mass was performed which was successful with no complications. Two days after the surgery, the patient reported that his vision was improving. Post-op MRI showed postsurgical changes with no definite residue and no acute complication (Figure 2). Histopathology exam showed pituitary gland tissue (most likely pituitary adenoma) with extensive necrosis and hemorrhage.

On the ophthalmologic exam 4 days after the surgery, visual acuity was 20/40 in both eyes which was at his baseline. There was no relative afferent pupillary defect. Extraocular movements were normal in both eyes. Ophthalmoscopy was bilaterally unremarkable with normal optic disc appearance. Visual field by confrontation technique was full in both eyes.

Postoperatively, the patient regained his normal mental status with no further episodes of confusion. Level of ACTH and endogenous cortisol remained low until the last follow-up, 9 months after the surgery (Table 1). The patient was found to be hypothyroid 9 months after the surgery. His testosterone level appeared to be low on 9 months, and his LH level dropped below the normal range, suggesting secondary hypogonadism. FSH and IGF-1 levels decreased to almost half while still being in the normal range (Table 1). The above-mentioned lab data indicates that the patient developed progressive hypopituitarism after the surgery.

## 3. Discussion

The clinical features of PA are typically sudden in onset and include headache, nausea/vomiting, visual disturbances, ophthalmoplegia, and altered consciousness [15].

Headache is present in more than 80% of the patients with pituitary apoplexy (PA) and is generally the initial manifestation [2]. PA typically causes an acute onset of severe frontal or retroorbital headache [16]. In a retrospective study on pituitary apoplexy cases, 95% of the patients presented with headache [17]. The potential mechanisms underlying headache in PA are meningeal irritation, dura-mater compression, enlargement of the sella turcica walls, or involvement of the superior division of the trigeminal nerve inside the cavernous sinus [1].

Visual disturbances are present in more than half of the patients with PA due to the compression of optic chiasm or optic nerves [2]. Variable degrees of visual-field impairment may be observed, but blindness is rare [2]. More than half of patients with PA have ocular motor palsy, due to functional impairment of CN III (the most affected), CN IV, and/or CN VI [2]. Nausea and vomiting may occur in PA due to meningeal irritation, adrenal insufficiency, hypothalamic dysfunction, or raised intracranial pressure [1].

Altered LOC is the most severe neurological finding in patients with pituitary apoplexy [18]. It is seen in around 20% of PA patients, and may range from mild lethargy to stupor and coma [19]. The mechanism of altered LOC remains elusive and might be related to subarachnoid hemorrhage, increased intracranial pressure, obstructive hydrocephalus, adrenal insufficiency leading to arterial hypotension and/or hypoglycemia, and hypothalamic compression [1].

Our patient developed recurrent brief episodes of altered LOC. Clouding of consciousness with transient brief episodes of confusion has not been previously described in cases of PA. The exact cause of the altered LOC could not be clearly determined in our patient but it could be possibly caused by a transient episode of increased intracranial pressure, cerebral vasospasm, or seizure episodes. Compression of the vascular structures or vasospasm can rarely cause reversible or irreversible cerebral ischemia in pituitary apoplexy cases [20]; while MRI did not indicate a compressive effect on the vascular structures, vasospasm could have happened. Subarachnoid hemorrhage was ruled out in our case, based on the results of the CT scan and CSF analysis. Adrenal insufficiency would be less likely to cause episodes of altered LOC, as the episodes were not associated with hypotension or hypoglycemia, and hyponatremia was only mild.

In our case, CT scan, which was a conventional CT without coronal/sagittal reconstruction, did not show any findings suggestive of pituitary apoplexy while MRI demonstrated the pituitary mass and hemorrhage. Although CT scan may be used as a rapid screening test, in these patients, fine-cut CT scans, with and without contrast and with coronal and sagittal reconstructions, are necessary [16]. Compared to CT scan, MRI of the sella is superior in detecting tumor and hemorrhage/infarct [16]. MRI can also estimate the time of onset of hemorrhage and detect the superior and lateral extension of the tumor [16]. CT scan may show the intrasellar mass and hemorrhagic components in 80% of cases, but it is most useful in the acute setting (24–48 hours); after this time, blood intensity decreases and may be difficult to detect [2].

While bleeding inside the pituitary gland may occur without causing any symptoms [1], a typical pituitary apoplexy can have a catastrophic clinical manifestation. Based on what we observed in the present case and the review of the literature, the cases of pituitary apoplexy who initially present with ophthalmoplegia, as the only symptom or the main symptom, have a more chronic onset and probably a less dramatic clinical course. A relatively slower expansion of the mass may allow it to extend into the cavernous sinus and compress the elements inside it, without significant suprasellar extension and without causing acute neurologic events that would prompt immediate medical attention.

Common differential diagnoses of PA are subarachnoid hemorrhage and bacterial meningitis; other conditions include midbrain infarction, cavernous sinus thrombosis, migraine, hemorrhagic infarction in a Rathke's cleft cyst, and aneurysms [16, 21].

The first intervention after PA diagnosis is hemodynamic stabilization, correction of electrolyte disturbances, and corticosteroid administration [21]. After collecting blood sample for hematological, biochemistry, and hormonal analysis, glucocorticoids should be administered in supraphysiological doses not only to serve as replacement for endogenous hormone deficiency but also to help control the effect of edema [1].

Regarding endocrine deficiencies, signs and symptoms of hypocortisolism are generally observed in the early stage after apoplexy onset [22], as occurred in our patient. Hypothyroidism, hypogonadism, and growth hormone deficiency are extremely frequent and may occur progressively during weeks, months, or years [22]. Therefore, a periodic reevaluation of anterior pituitary function is recommended [22]. It has been suggested that pituitary deficiencies, once established, usually do not recover, regardless the treatment [2, 21]. Patients with low levels of prolactin exhibit a lower probability of pituitary function recovery after surgery [21, 23]. Hyponatremia, observed in 40% of cases, can be secondary to hypocortisolism or inappropriate antidiuretic hormone secretion [21].

This case report illustrates an unusual clinical presentations of PA: the patient had pupil-sparing CN III palsy, which is more characteristic of ischemic rather than compressive causes; CN IV palsy is not common in PA cases; and altered LOC in PA patients is usually associated with headache [24], nausea, vomiting, signs of meningismus, or evidence of subarachnoid hemorrhage none of which was present in our patient. There are some case reports that illustrated even more unusual or rare presentations of PA, such as PA presenting as bilateral anterior cerebral artery infarction [20] or diabetic ketoacidosis [25, 26]. The importance is to recognize these atypical presentations so that definitive therapy can be instituted.

Other than the wide variety of clinical presentations of PA, one should bear in mind that apoplexy is rare and can frequently develop before the diagnosis of pituitary adenoma is made. Moreover, CT scan cannot detect all the cases of pituitary apoplexy, especially if CT is obtained after the first 48 hours of the onset of hemorrhage, as happened in our case. Therefore, a high index of suspicion is needed for the early diagnosis of this condition. The timely diagnosis and management of PA can improve the visual outcome, relieve the bothersome symptoms, accelerate the recovery of ophthalmoplegia, and prevent the potentially severe consequences of hormonal disturbances or the mass effect. This can significantly improve the care of our patients.

## 4. Conclusions

Pituitary apoplexy can present with ophthalmoplegia and altered level of consciousness (LOC) without exhibiting any signs or symptoms of increased intracranial pressure, or any evidence of subarachnoid hemorrhage. In this case of pituitary apoplexy, we observed clouding of consciousness exacerbated by transient brief episodes of confusion for which we could not find any immediate cause. PA can present as various clinical pictures and therefore mimic different diseases or pathologies. A high index of suspicion is required for early diagnosis and appropriate treatment of this potentially fatal disease.

## Figures and Tables

**Table 1:** Laboratory data findings.

Variable	Reference range	Day 1^*∗*^	Day 2	Day 3	Day 4	Day 5	Day 6	Day 7	Day 8	Day 9	Day 10	3 months	6 months	9 months
Glucose, random (mg/dL)	74–106	103	90	83	84	89	115	118	107	109	84			84

Na, serum (mEq/L)	136–145	130		131	130									

K, serum (mEq/L)	3.6–5.0	4.0		4.3	4.3									

Thyroid stimulating hormone (mU/L)	0.40–4.50							0.114						1.68

Thyroxine (T4), free (ng/dL)	0.76–1.46			0.92							1.02	1.0		0.7

Triiodothyronine (T3), total (ng/dL)	76–181				61									

Follicular stimulating hormone (mIU/m)	1.6–8			6.4				4.8				3.1		

Luteinizing hormone (mIU/m)	1.6–15.2			2.8				3.2				1.3		1.3

Testosterone (ng/dL)	250–1100													14

Prolactin (ng/mL)	2.0–18.0			<1.0							<1.0	<1.0		<1.0

ACTH (pg/mL)	6–50			<5				<5				<5		<5

Serum osmolality (mosm)	274–309		273		288				298					

Urine osmolality (mosm)	281–1076				468				403	569				

AM Cortisol (mcg/dL)	4.0–22.0				1.1, 5.2, 11.6^*∗∗*^									0.9^*∗∗∗*^

Insulin growth factor-1 (ng/mL)	34–245				71			75				36		

^*∗*^Day 1 is the first day of admission at our hospital. ^*∗∗*^Cortisol level was 1.1 mcg/dL at 8 am before stimulation. It was 5.2 mcg/dL and 11.6 mcg/dL after 30 minutes and 60 minutes of cosyntropin injection, respectively. ^*∗∗∗*^The patient was told to skip the last two doses of steroid before this measurement.
